# A semi-empirical approach to calibrate simulation models for semiconductor devices

**DOI:** 10.1038/s41598-023-36196-z

**Published:** 2023-06-27

**Authors:** Rahul Jaiswal, Manel Martínez-Ramón, Tito Busani

**Affiliations:** 1 CHTM, University of New Mexico, 1313 Goddard St SE, Albuquerque, NM 87106 USA; 2 ECE, University of New Mexico, 498 Terrace St NE, Albuquerque, NM 87106 USA

**Keywords:** Solar energy, Scientific data, Electrical and electronic engineering, Computational science

## Abstract

Semiconductor device optimization using computer-based prototyping techniques like simulation or machine learning digital twins can be time and resource efficient compared to the conventional strategy of iterating over device design variations by fabricating the actual device. Ideally, simulation models require perfect calibration of material parameters for the model to represent a particular semiconductor device. This calibration process itself can require characterization information of the device and its precursors and extensive expert knowledge of non characterizable parameters and their tuning. We propose a hybrid method to calibrate multiple simulation models for a device using minimal characterization data and machine learning-based prediction models. A photovoltaic device is chosen as the example for this technique where optical and electrical simulation models of an industrially manufactured silicon solar cell are calibrated and the simulated device performance is compared with the measurement data from the physical device.

## Introduction

A semiconductor device can be modeled using a set of information like device physics, device structural parameters, material properties in the device, and external stimulus required.

Such a simulation model can be formulated with literary information. However, a single device technology can have multiple variations, this can result not only from changes in device architecture but also due to process variations from individual tools in a lab or production line, leading to deviation of material properties from data available in the literature.

These variations require tuning the parameters used in a standard simulation model to match the characteristics of a specific device variation. Some design parameters can be measured easily from the fully integrated device and can be plugged into the simulation model directly. However, there can be parameters that either cannot be measured at all or directly using a characterization tool. There can also be a set of material parameters required for the simulation model of the semiconductor device that cannot be measured from the fully integrated device and instead require a precursor of the device or even a device variation that is not part of the fabrication recipe. One example of such a case can be the minority carrier lifetime^1^ in a solar cell device that has a direct impact on the final power conversion efficiency and can be used for performing loss analysis. Minority carrier lifetime is a quantity that can be directly measured using QSSPC (Quasi steady-state photoconductance)^1^, but a symmetric sample is required, instead of a fully integrated solar cell device which is an asymmetrical device composed of different kinds of layers for electron and hole charge extraction. A lifetime sample is not a precursor for the final solar cell, and it has to be manufactured just for the measurement of the minority carrier lifetime.

The issue of variation in material parameters and its impact on designing a device simulation model has been discussed in the literature. Madan et al.^2^ pointed out that refractive indices for the same material in a perovskite solar cell can vary due to different fabricating recipes and emphasized validating simulated results with experimental findings. Giesl et al.^3^ have emphasized how calibration of a simulation model may require a number of parameters that can not be experimentally measured. Zeman et al.^4^ also discusses the issue of material parameter variation and provides a conventional approach to calibrate simulation models using parameter fitting. Rose et al.^5^ provide emphasis on how precise device physics modeling is required for calibrating simulation models to match simulated and measurement data.

Precise calibration of simulation models is also important as efforts are being made to create digital twins^6^ for a semiconductor device using machine learning (ML) techniques. Instead of using measurement data, simulated models that are calibrated are preferred as they scale better in terms of time and resources required to create a sufficient amount of training data required for a learning model. This is possible as once the simulation model is calibrated, input parameters can be varied to prototype device variations, doing so, with fabricating and characterizing physical devices will be inefficient. Buratti et al.^7^ proposed a methodology to extract bulk defect parameters in silicon by using machine learning models trained with simulation data, they also pitched the idea of transfer learning where the same methodology can be applied to other materials. Mohnsen and Altermatt^6^ used digital twins for Passivated Emitter and Rear Cell (PERC)^8^ solar cells, these digital twins are machine learning models trained with TCAD simulation^9^ data for identifying how variations in material and device design parameters can affect the PERC solar cell performance, and their findings included that a particular performance metric can result from more than one combination of material and device parameters set. Kaya and Hajimirza^10^ also show that a black box approach of using ML models to optimize device performance is efficient compared to fitting a numerical simulation model, the emphasis was not on creating a perfectly calibrated simulation model but to create a pool of data set by varying individual parameters and predicting device performance to identify the optimized device characteristics. The concept of using machine learning techniques to also optimize a processing recipe was demonstrated in Mohnsen et al.^11^.

Our proposed methodology is based on the hypothesis that ML models can learn from a minimal data set and can try to predict a target value by interpolating or extrapolating the training data points. In contrast to contemporary works in literature, the focus of this work is to perform calibration of more than one simulation model for a semiconductor device for predicting more than one performance metric. There are semiconductor devices like solar cells, where the optical and electrical parameters are generally simulated separately, the electrical simulation model is reliant on the data from the optical simulation model, and they share a common set of device and material parameters, therefore calibration of electrical simulation models is dependent on calibration of optical simulation models. This is a challenging task as generally literary works have tried to optimize one performance metric of a device at a time, which can overlook the trade-offs that exist in a device, a narrow example of it can be the trade-off when designing the thickness parameter of metal fingers, increasing it will reduce the resistance faced during charge extraction, while simultaneously decreasing the area on which light can fall, therefore reducing the optical generation within the device.

We chose Gaussian process regression (GPR)^12^ as the ML methodology for this work, mainly because of two reasons: One, most relationships between semiconductor devices, material, and performance parameters are nonlinear in nature, and because GPR’s use kernel functions^13^ $$\phi (\cdot )$$ to map input features into a higher dimensional Hilbert space endowed with a dot product $$K(\textbf{x}_i,\textbf{x}_j)=\phi ^{\top }(\textbf{x}_i)\phi (\textbf{x}_j)$$ between two points *i* and *j*. These nonlinearities are learned by the machine learning model for the multidimensional input feature set. Another advantage of using the GPR is that we can evaluate the variance terms composed of the kernel inner product of the training points, between the training point and the test point, and of the test points. Behaviorally these are the variance in the training or test data set (*K* and $$K_{**}$$ respectively), and the covariance between the test and training data set ($$K_*$$). We can construct a kernel covariance matrix ($$\Sigma $$) using these variances and covariances, which provides the knowledge of variance within every prediction of the ML model.

$$\begin{aligned} \Sigma = \begin{bmatrix} K &{} . &{} K_*\\ . &{} . &{} . \\ {K_*}^T &{} . &{} K_{**} \end{bmatrix} \end{aligned}$$

“Target device” Section discusses the target device, the performance metrics associated with the device and the simulation models used for the device. “Proposed Methodology” Section discusses the proposed methodology of multi-model calibration. “Results” Section presents the results and “Discussion” Section is dedicated to the discussion of the results and conclusion of the proposed work.

## Target device

**Figure 1:**
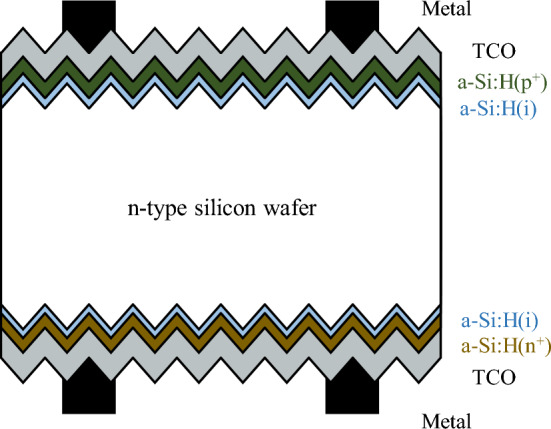
HIT solar cell band architecture.

The target device for this work is the silicon heterojunction solar cell (HIT)^14^. The heterojunction with thin intrinsic layer architecture is shown in Fig. 1.

The operation of a silicon solar cell can be generalized by three mechanisms: charge generation, charge separation, and charge extraction. A solar cell device is structured to maximize the efficiencies of these three mechanisms, HIT cells achieve this by implementing a hetero-structure between amorphous and crystalline silicon.

HIT solar cells also have some inherent advantages over other commercial technologies like PERC solar cells, for example, lower temperature coefficient for power conversion efficiency. Commercialization of HIT cell technology is increasing, but a significant amount of research is still needed to mitigate issues like degradation of open circuit voltage ($V_{OC}$)^15^ over time. Simulation and machine learning-based device prototyping can expedite the process of optimizing a HIT solar cell device.

For this research work, a commercially available TCAD tool suite (Sentaurus^9^) was used for designing simulation models. An important parameter for the optical simulation will require the complex refractive index profile^16^ for the transparent conductive oxide (TCO)^17^ layer which also acts like anti-reflective (ARC)^18^, we can measure it using techniques like ellipsometry^19^, but it will require a highly polished (single-side) high-resistivity silicon wafer with (singe-side) TCO layer (deposited on a polished surface). Such a sample is not a precursor to an industrially fabricated solar cell and would require disruption of the pilot line, henceforth a baseline complex refractive index is taken from the literature and optimized for the device using machine learning methods. Ray tracing and transfer matrix methods^20^ are used to calculate the absorbed photon density, and texturing using inverted pyramids was specified in the simulation structure. The reflection profile^19^ and optical generation profile^21^ can be obtained using the optical simulation model. The goal is to calibrate the optical simulation model by matching the reflection profile to the measured profile as the optical generation profile of a solar cell cannot be directly measured.

The power conversion efficiency of a solar cell device is calculated from its current–voltage profile. The electrical simulation model for the current–voltage profile requires information about the optically generated carrier (electron-hole pairs) density across the density, which is obtained from the optical simulation model. Other device mechanisms like carrier recombination, charge separation, and extraction also govern the evaluation of the current–voltage profile in the simulation model.

## Proposed methodology

A generalized algorithm to calibrate different semiconductor devices had to be considered, to prototype a device, multiple simulation models can be required instead of a single simulation, our goal is to calibrate these multiple simulation models at a time in order to compensate for any trade-off that might result from variations in parameters that are common to multiple simulation models. In “Individual model calibration” Section, the methodology to calibrate one simulation model is presented, and in “System design” Section, the methodology to integrate the calibration process of multiple simulation models into a single system is presented.

For our target device, we start with performing characterization on the device and its precursors to establish the data set for the different optical and electrical characteristics of the device, this includes characterizing the current–voltage profile, quantum efficiency (QE)^22^, reflection profile, and minority carrier lifetime profile of the HIT cell. We are using a ML based model in the calibration algorithm, although it should not be confused with any ML based prediction models mentioned in the text, where the goal is to prototype device performance. Throughout the text, any simulation described refers to a TCAD simulation. ML model based calculations are explicitly referred as predictions.

### Individual model calibration

To start the calibration process for a simulation model, baseline simulations are required. An initial value range (from literature or provided by the device manufacturer) for every simulation input parameter that needs to be calibrated is assigned. Parameters that are known with absolute values (measured data or data provided by the manufacturer) will not be part of the calibration process, and ‘input parameters’ from now on refer to parameters that need to be calibrated unless explicitly mentioned otherwise. It is a good practice to remove redundant parameters from the set of parameters targeted for calibration (i.e. parameters that are highly correlated).

An input parameter grid is created by varying the input parameters in their assigned range This range can be taken from literature (provided in Table 1 for our work) and the subdivision can be arbitrary, but expert knowledge will be helpful in choosing optimal subdivisions. For *n* input parameters and value ranges $[(x_{1}^\text {initial}, x_{1}^\text {final}),(x_{2}^\text {initial}, x_{2}^\text {final}) \ldots (x_{n}^\text {initial}, x_{n}^\text {final})]$ with different number of steps $[s_1, s_2, s_3\ldots s_n]$, a total of $S_1 = \prod _{i=1}^{n} {s_1, s_2, s_3 \ldots s_n}$ simulations will be run.

These $S_{1}$ baseline simulations are used to train the GPR models, where input parameters of the simulation models are the input features of the machine learning model, and the simulated data is the prediction target.

Then the input parameter of $S_{1}$ data points (each data point referring to one combination of input parameters) is interpolated to get a finer observation between device parameters and the corresponding performance characteristics. A total of three values are calculated using interpolation between each adjacent value of an input parameter, which are equidistant. Therefore a total of $S_{2} = 4S_{1}\;-\;3$ data points are created.

The $S_2$ interpolated data points grid can now be used for making predictions from the trained machine learning model. Predicting the device performance is much more efficient in terms of resources and time required compared to simulating the device performance at this scale.

Since most characteristics in a semiconductor device are characterized as a profile (for example, the current–voltage profile in a solar cell is a list of current values at different voltage values), predictions from the machine learning model are also targeted to create a profile by combining different point predictions (for example, by combining current value predictions for different voltage value points, keeping other parameters the same). Each prediction profile is compared against the corresponding measured profile, and then the r-squared (R2)^23^ score between each prediction profile is calculated to determine the prediction accuracy.

If the condition ‘C1’ : “a prediction profile exists where the R2 score is more than 90% and 80% of the measured values lie within the 95% confidence bound (2 standard deviations) of the prediction” is ‘True’, that particular prediction is chosen as a calibrated prediction and the corresponding input parameter set is declared as the calibrated input parameter set.

**Figure 2:**
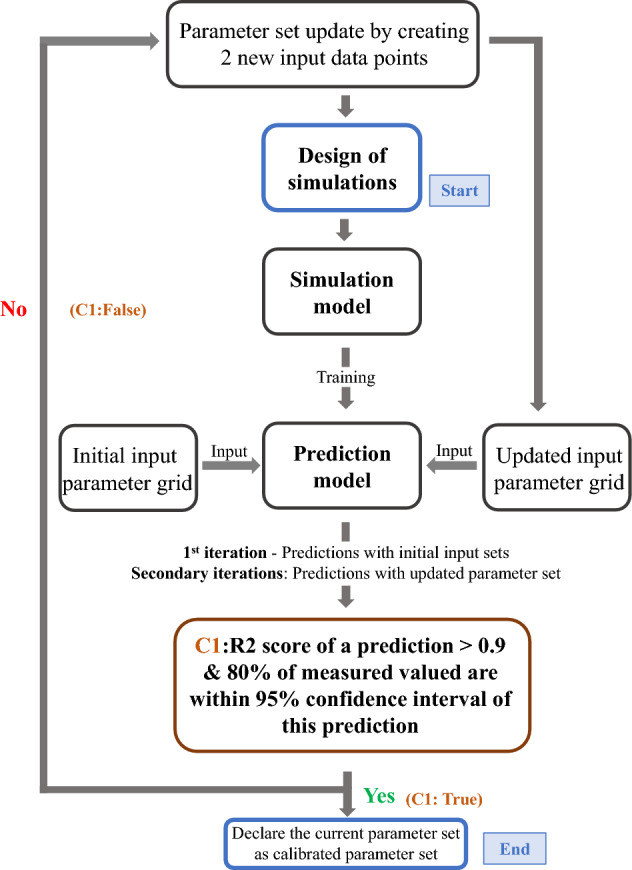
Process flow to calibrate a single simulation model.

If condition ‘C1’ is ‘False’, the prediction with the highest R2 score is chosen and the input parameters of this particular prediction are taken as baseline values. Additional parameter sets are created by varying the parameter values by ± 5%. These two new sets of values are used to do additional simulations and the ML models are retrained with the updated simulation data. The $S_{2}$ input parameter grid values are updated with these two new sets of values and are interpolated again. Predictions from the ML models with updated input parameter sets are compared against the measured values to check condition ‘C1’. This process of updating the parameter set and training data was referred to as ‘revision’ in our work and the whole process is repeated until the goal is either achieved or a maximum number of attempts are performed (set as 500 by us to not overflow the computational resources). In the former case, the calibration process is successful and manual intervention is required for the latter case. This algorithm flow is shown in Fig. 2.

The second part of the condition ‘C1’ is established to make sure that the machine is confident in its predictions, instead of just relying on the accuracy score.

### System design

**Figure 3:**
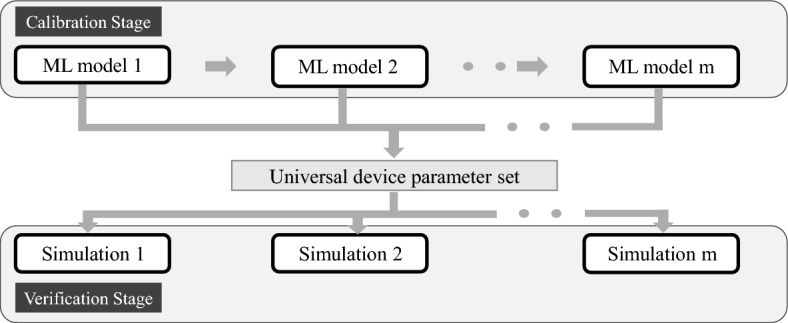
Process flow to calibrate a system of simulation models.

The operation of a semiconductor device can be explained using several mathematical or physical models, this is the reason why multi-physics modeling tools are generally used to simulate the complete device behavior. Similarly, the strategy implemented in our work was to calibrate individual simulation models in a hierarchical fashion, such that if an electrical simulation model is dependent on the results of an optical simulation model, calibration of the optical simulation is done prior to the electrical model, and the data from the calibrated optical simulation model is provided as one of the inputs to the model electrical simulation model calibration. Fig. 3, visualizes this strategy.

Once all the simulation models are calibrated independently, the calibrated parameters from each of these simulations are pooled into a universal parameter dataset. Parameters that are common between multiple simulation models are averaged together in our work, but a more optimal way will be to use a weighted average (based on how sensitive a performance metric is to a given material/device parameter). This universal parameter dataset is then used to provide inputs to all the simulation models to verify that the simulated results are 90% accurate compared to the measurement data.

We applied the concept presented in Fig. 3 to the HJT cell and divided the cell into the optical and electrical domains. Indeed two simulation models were calibrated. One was the optical simulation model that calculates the optical generation profile and the reflection profile. The second was the simulation model to calculate the current–voltage profile of the device under AM1.5G^24^ illumination. Two additional simulation models to calculate the QE and minority carrier lifetime profile were not calibrated (as they use the same input parameters as the current–voltage simulation model), instead, they were used during the verification, to check if their simulated output was at least 90% accurate to the corresponding measurement data.

## Results

The list of parameters in electrical and optical simulations of the HIT solar cell is provided in Table 1. Substrate thickness was 160 μmf after etching. Thicknesses of the amorphous silicon layers: intrinsic layer (a-Si), n-doped layer ($a-Si(n^+)$) and p-doped layer ($a-Si(p^+)$) were provided by the cell manufacturer. $D_{it}$ represents interface trap density.

**Table 1:** Final (calibrated) list of parameters.

Parameter	Initial value	Calibrated value	Unit
$a-Si(p^{+})$ doping	^25,26^	2.3e19	${\rm cm}^{-3}$
$a-Si(n^{+})$ doping	^25,26^	5.75e18	$\textrm{cm}^{-3}$
SRV for silicon-Silver SRV for silicon-Aluminium	^27^	5.7e4	$\textrm{cm}/\textrm{s}$
Contact resistivity	^28,29^	8.9e-3	$\Omega \textrm{cm}^{2}$
$D_{it}$ at c-Si - a-Si interface	^30,31^	1.2e11	$\textrm{cm}^{-2}$
Trap density states $a-Si(n^{+})$	^32^	9e18	$\textrm{cm}^{-3}$
TCO Complex refractive index	^33^	Supplementary information	
TCO thickness	^34,35^	71.25	$\textrm{nm}$
Wafer resistivity	0.6–1.2 (Provided by manufacturer)	0.6312	$\Omega \;\textrm{cm}$
Bulk lifetime	3–7 (Provided by manufacturer)	3.3	$\textrm{ms}$

Indium Tin Oxide (ITO) was used as the TCO layer, acting as an ARC coating layer for the solar cell. The complex refractive index values (n-k data) were taken from literature^33^ and were calibrated for the optical simulations. These n-k pair of values were interpolated at a fixed number of wavelength values as they were input parameters to the ML model. Partial information about the calibrated complex refractive index is provided in the supplementary information.

**Figure 4:**
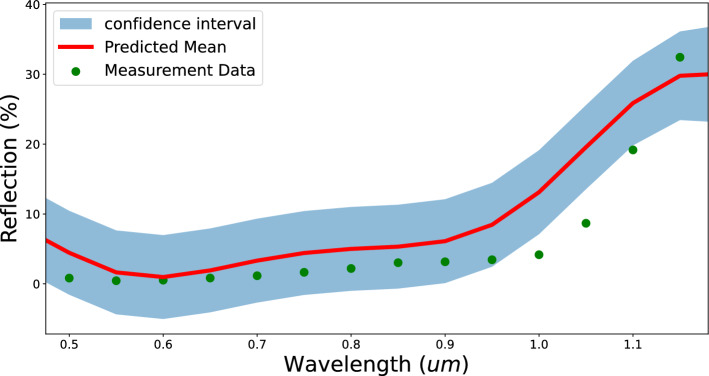
Predicted reflectance profile compared to measured profile.

Substrate doping was calculated from the wafer resistivity value range provided by the manufacturer, and bulk lifetime^36^ was also provided as a value range by the cell manufacturer. The calibration of the optical simulation models required 2 additional revisions in addition to the initial parameter grid and training data for the ML model.

**Figure 5:**
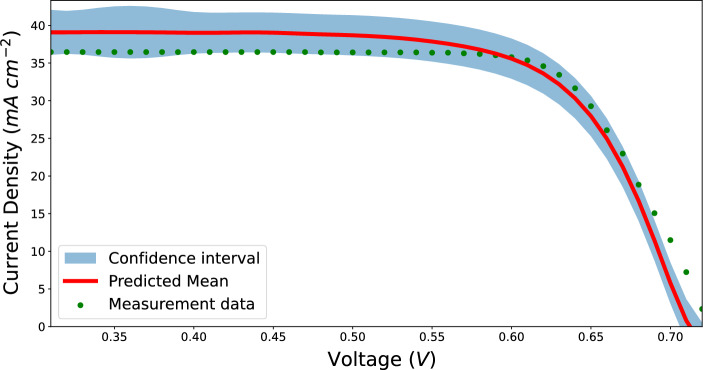
Predicted current–voltage profile compared to measured profile.

**Figure 6:**
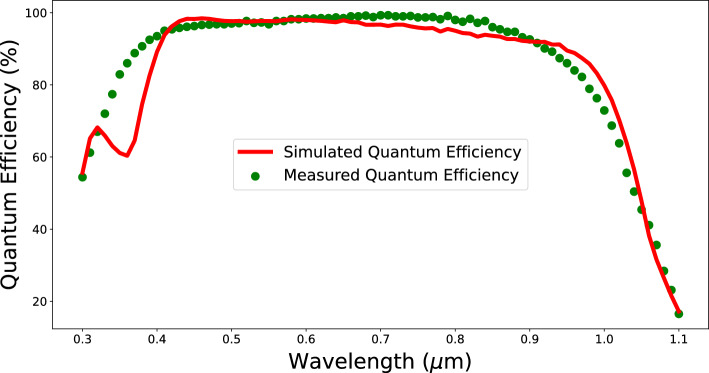
Simulated quantum efficiency profile compared to measured profile.

**Figure 7:**
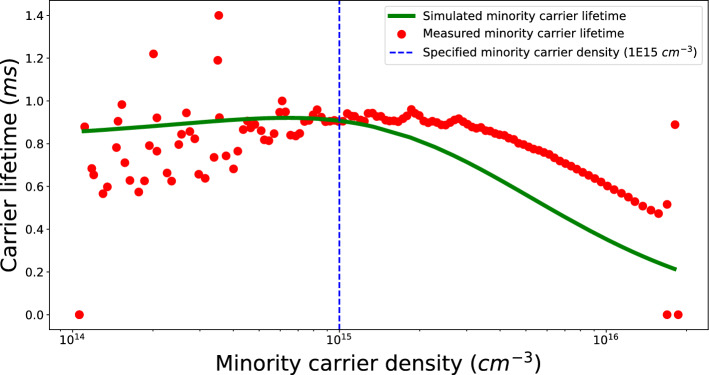
Simulated minority carrier lifetime profile compared to measured profile.

Calibration of the current–voltage profile simulation model required one revision in addition to the initial parameter grid and training data for the ML model. Once both optical and current–voltage simulation models were calibrated individually, the common parameters between them were averaged together to create a universal list of calibrated parameters for this solar cell provided in Table 1. Predictions for the reflectance profile and current–voltage profile using the calibrated parameters are compared against the measurement data from the precursor and the final solar cell in Figs. 4 and 5 respectively. The confidence intervals for each of these predictions are also shown. The short circuit current was lower in the measurement compared to the predicted value due to the imperfect contact (the fully contacted samples had bus strings instead of bus bars), but this comparison shows that the ML model is capable to compensate for measurement artifacts/errors.

During the verification of the calibrated parameters, QE and minority carrier lifetime profiles were also simulated using the calibrated parameter list and are shown in Figs. 6 and 7 respectively.

For training using the GPR model, the kernel function was scaled with a constant mean, a standard Gaussian likelihood^37^ function (where all inputs have the same observational noise) was used and Adam optimizer^38^ was used for loss analysis. 540 data initial data points were used for training the ML model for optical simulation model calibration and 1426 data points were used for training the ML model for current–voltage simulation model calibration.

## Discussion

The crux of this research is to perform calibration of simulation models with minimal resources, which was achieved for an industrially manufactured HIT solar cell. One key aspect that is unique to this work is the minimal amount of measurements and simulation data required for training a ML model , a ML model inherently requires a larger dataset to learn and estimate device performance compared to expert knowledge-based calculation, henceforth this act of data generation should be efficient. The initial parameter set used for creating initial simulation data was not evenly spread, as instead of performing a grid search for parameters, the proposed methodology looks for the best parameter fits in every iteration using not just the accuracy scores but also the confidence of predictions. This is not a true Bayesian optimization^39^ approach in a strict sense, as parameter tuning is not done based on a global optimum search using an acquisition function with a risk exploration to return reward-based strategy. This is not crucial as there is no need to search for a global optimum parameter, given that semiconductor device parameters have to be constrained within a range (provided by the manufacturer, material properties, and device physics). The proposed methodology keeps the number of additional simulations required (in addition to the initial simulations) for training by looking for the best parameter fit in an iteration, and the majority of fitting work is done by the ML model predictions which are exponentially faster (within seconds), compared to a TCAD based simulation, which can take hours.

Another key aspect of the proposed strategy is the flexibility of configuring the design, for example, the interpolation between simulation data points to create a prediction parameter grid can be tuned by increasing or decreasing the number of interpolated points between two adjacent simulated data points. This algorithm can be used to detect redundant parameters that are being calibrated because a set of dependent parameters will be linearly varying during the calibration process.

One limiting factor that can have an effect on the efficiency of this proposed methodology is the quality of characterization data, for example, the sun simulator-based I–V tester used for measuring the current–voltage response from the fully manufactured device has some degradation in its contacts, adding parasitic resistances, this will in-turn make the calibration system compensate for parameter unnecessarily. The QSSPC tool used for measuring minority carrier lifetime in the cell precursor (without metal contact grids) added measurement artifacts in the data, making a direct accuracy score redundant, instead, an accuracy check was done near a specified minority carrier density value of 1.0e15 carriers $\textrm{cm}^{-3}$. Another potential limiting factor can be the optimization of the ML model itself, bias-variance trade-offs^40^ have to be optimized in order to make sure that the ML models are not over or under-trained for a given training dataset. One observation made during the calibration of HIT solar cell’s optical simulation model was the variance when predicting reflection profile as multiple combinations of input parameter sets can provide very close prediction values, the ML model was inherently less confident near certain wavelength values.

## Conclusion

This proposed methodology provides an innovative way to fine-tune input parameters for multiple simulation models of a semiconductor device, even for cases when simulation models can be interdependent. While expert knowledge of the device is useful to set boundary values (ranges) for parameter values that need calibration, using data-based learning reduces the pure brute force nature required in the conventional way of calibrating a simulation model using simulation and experiments only. Although an argument can be made for the proposed methodology that it is a black-box approach, the main task of the proposed algorithm is to find correlations between the device and material parameters and device characteristics, rather than emulating device physics using ML, like the strategy implemented in digital twin designs, making the proposed approach generalized for multiple use-cases.

The proposed methodology can be expanded for other semiconductor devices, it can also be used for calibrating process simulations for optimizing device fabrication recipes.

## Supplementary Information

Supplementary Information.

## Data Availability

Available on reasonable request to the corresponding author.
