# Supplementary material for: A semi-empirical approach to calibrate simulation models for semiconductor devices
Source: Sci Rep. 2023 Jun 27;13:10436. doi: 10.1038/s41598-023-36196-z (PMC10300133; doi:10.1038/s41598-023-36196-z)
Supplement: Supplementary file 1 — Supplementary Information. [file 41598_2023_36196_MOESM1_ESM.pdf]

Supplementary information

Table 1 provides the calibrated complex refractive index for the Transparent Conductive Oxide (TCO) layer. This data is interpolated at specific wavelength values for data integrity.

Table 1 Interpolated complex refractive index for TCO at specific wavelengths

**wavelength (μm)**	**n**	**ik**
0.3	2.154767	0.175205
0.6	1.88984	0.00239
0.9	1.618321	0.014275
1.2	1.402621	0.0356749999999999
